# Tethered homing gene drives: A new design for spatially restricted population replacement and suppression

**DOI:** 10.1111/eva.12827

**Published:** 2019-06-17

**Authors:** Sumit Dhole, Alun L. Lloyd, Fred Gould

**Affiliations:** ^1^ Department of Entomology and Plant Pathology North Carolina State University Raleigh North Carolina; ^2^ Biomathematics Graduate Program and Department of Mathematics North Carolina State University Raleigh North Carolina; ^3^ Genetic Engineering and Society Center North Carolina State University Raleigh North Carolina

**Keywords:** Cas, CRISPR, gene drive, genetic engineering, population alteration, underdominance

## Abstract

Optimism regarding potential epidemiological and conservation applications of modern gene drives is tempered by concern about the possibility of unintended spread of engineered organisms beyond the target population. In response, several novel gene drive approaches have been proposed that can, under certain conditions, locally alter characteristics of a population. One challenge for these gene drives is the difficulty of achieving high levels of localized population suppression without very large releases in the face of gene flow. We present a new gene drive system, tethered homing (TH), with improved capacity for both localization and population suppression. The TH drive is based on driving a payload gene using a homing construct that is anchored to a spatially restricted gene drive. We use a proof‐of‐concept mathematical model to show the dynamics of a TH drive that uses engineered underdominance as an anchor. This system is composed of a split homing drive and a two‐locus engineered underdominance drive linked to one part of the split drive (the Cas endonuclease). We use simple population genetic simulations to show that the tethered homing technique can offer improved localized spread of costly transgenic payload genes. Additionally, the TH system offers the ability to gradually adjust the genetic load in a population after the initial alteration, with minimal additional release effort. We discuss potential solutions for improving localization and the feasibility of creating TH drive systems. Further research with models that include additional biological details will be needed to better understand how TH drives would behave in natural populations, but the preliminary results shown here suggest that tethered homing drives can be a useful addition to the repertoire of localized gene drives.

## 1 INTRODUCTION

The development of population genetic theory related to use of translocations and other underdominance mechanisms to suppress pest populations or change their characteristics started more than seven decades ago (Curtis, 1968; Serebrovsky, 1940; Vanderplank, 1947). Under the best of circumstances, these approaches were expected to require release of large numbers of genetically manipulated individuals, and would have only localized impacts. Despite major efforts, early empirical attempts to use these approaches were unsuccessful (Gould & Schliekelman, 2004). Advances in transgenic techniques for engineering insects spurred the hope that natural populations of insect pests could be suppressed or manipulated using transposable elements (O'Brochta et al., 2003; Ribeiro & Kidwell, 1994) and other types of selfish genetic elements (Sinkins & Gould, 2006). The expectation was that fewer individuals would need to be released and the spread would not be localized (Burt, 2003). In spite of the slow initial progress with such approaches (Carareto et al., 1997; Chen et al., 2007; Windbichler, Papathanos, & Crisanti, 2008), researchers and popular media commentators raised concerns that unrestricted spread could be problematic and that safeguards were needed (Burt, 2003; Gould, 2008). With the recent development of CRISPR/Cas‐based gene drives (Esvelt, Smidler, Catteruccia, & Church, 2014; Gantz & Bier, 2015), such concerns have intensified and refocused attention on spatially and temporally restricted gene drives as safer alternatives for many applications (Akbari et al., 2015; Marshall & Akbari, 2018; Min, Smidler, Najjar, & Esvelt, 2018; NASEM, 2016).

A number of strategies have been put forth for gene drives that are expected to be relatively restricted either spatially (Akbari et al., 2014; Buchman, Ivy, Marshall, Akbari, & Hay, 2018; Davis, Bax, & Grewe, 2001; Marshall & Hay, 2012a; Oberhofer, Ivy, & Hay, 2019; Reeves, Bryk, Altrock, Denton, & Reed, 2014), temporally (Gould, Huang, Legros, & Lloyd, 2008), or both temporally and spatially (Burt & Deredec, 2018; Noble et al., 2016; Rasgon, 2009). Marshall and Hay (2012a) and Dhole, Vell, Lloyd, and Gould (2018) have compared some of the properties of these gene drive systems using simple population genetics models. Spatially restricted gene drives are generally not expected to establish themselves at high frequency in neighboring populations when migration rates are low (<1%; but see some results in Champer, Zhao, Champer, Liu, & Messer, 2018; Dhole et al., 2018). As migration rates to neighboring populations increase, spatial restriction to the targeted population is not assured (Champer, Zhao, et al., 2018; Dhole et al., 2018; Marshall & Hay, 2012a).

The release size required for different gene drives to successfully invade a population varies widely even when the drive constructs do not impose high fitness costs. If instead of population replacement, the goal of a release is population suppression, the drive constructs must impose high fitness costs, and because of that, the spatially confined drives require much larger releases to spread into a local population, if they can spread at all (Dhole et al., 2018; Edgington & Alphey, 2018; Khamis, Mouden, Kura, & Bonsall, 2018; Magori & Gould, 2006; Marshall & Hay, 2012a; Ward et al., 2011). In general, gene drives that require larger releases when the constructs have low fitness costs tend to remain more localized to the target population. However, these gene drives are also the least likely to be able to drive constructs with high fitness costs into the target population (Dhole et al., 2018; Marshall & Hay, 2012a).

There is a need for a gene drive that is reasonably confined and can spread constructs with high fitness costs. We recently described a spatially restricted gene drive for population suppression that relies on a CRISPR/Cas endonuclease that disrupts an allele that is fixed in the target population, but cannot disrupt other alleles at the same locus that are found at least at low frequencies in neighboring populations (Sudweeks et al., 2019). That approach would be specifically appropriate for small populations on oceanic islands where genetic drift is expected to be strong. Min, Noble, Najjar, and Esvelt (2017) have also outlined a verbal model of a gene drive construct, the daisy quorum drive that may potentially allow localized spread of high‐cost payloads with relatively small release size. A mathematical exploration of the dynamics of the daisy quorum drive is not yet available, but the design is a promising development.

Here, we propose a new concept of tethered homing (TH) gene drives. These drives include a homing component that does not drive on its own, but is “tethered” by engineering it to be reliant on a spatially restricted gene drive. We present the dynamics of a specific TH gene drive design that uses a two‐locus engineered underdominance component to tether a CRISPR/Cas‐based homing component (underdominance tethered homing, UTH). Conceptually, the homing component can instead be tethered to a different localized gene drive, such as one‐locus engineered underdominance, chromosomal translocations, or one of the poison–antidote systems (Akbari et al., 2013; Marshall & Hay, 2012b; Oberhofer et al., 2019; Reeves et al., 2014). The concept of combining different gene drive strategies is not new; Huang, Magori, Lloyd, and Gould (2007) have analyzed the possibility of using engineered underdominance drive with meiotic drive or with *Wolbachia* endosymbionts. Min et al. (2017) have proposed using a homing construct to drive an underdominance drive to high frequency in the population (conceptually an inverse of the TH concept which uses a spatially restricted drive to anchor a homing construct). The TH drive system is a split drive (Dicarlo, Chavez, Dietz, Esvelt, & Church, 2015; Esvelt et al., 2014) combined with a spatially restricted gene drive.

As with other analyses aimed at initial description of novel gene drive systems (Burt, 2003; Burt & Deredec, 2018; Davis et al., 2001; Gould et al., 2008; Marshall & Hay, 2012b), we explore the properties of the UTH strategy using a very general, proof‐of‐concept mathematical model (Servedio et al., 2014). In addition to describing the general dynamics of the UTH drive, our analyses are specifically intended to facilitate direct comparison of this drive with three previous gene drives designed for localized population alteration—the daisy‐chain drive (Noble et al., 2016) and two engineered underdominance drives (Davis et al., 2001). We demonstrate that a UTH gene drive can offer an improvement in localization level. A UTH drive can especially be useful to locally spread high‐cost payload genes while having relatively small effects on neighboring populations. Because of its ability to spread high‐cost payloads, the UTH drive should have greater potential for suppressing populations than the other three drives, but more detailed models and experiments will be needed to quantitatively assess the extent of this potential. We discuss possible improvements to the UTH drive design and propose another TH drive design based on the recent Cleaver/Rescue gene drive (Oberhofer et al., 2019).

## 2 MATERIALS AND METHODS

### 2.1 Drive design

The underdominance tethered homing (UTH) drive is a two‐component drive. The first component is a two‐locus engineered underdominance drive linked to genes for producing a Cas endonuclease. The second component is a construct containing sequences coding for multiple guide RNAs that, in the presence of the Cas endonuclease, target the wild‐type gene on the homologous chromosome, in turn triggering homing through the cell's homology‐directed repair (HDR) pathway (see details below).

**Figure 1:**
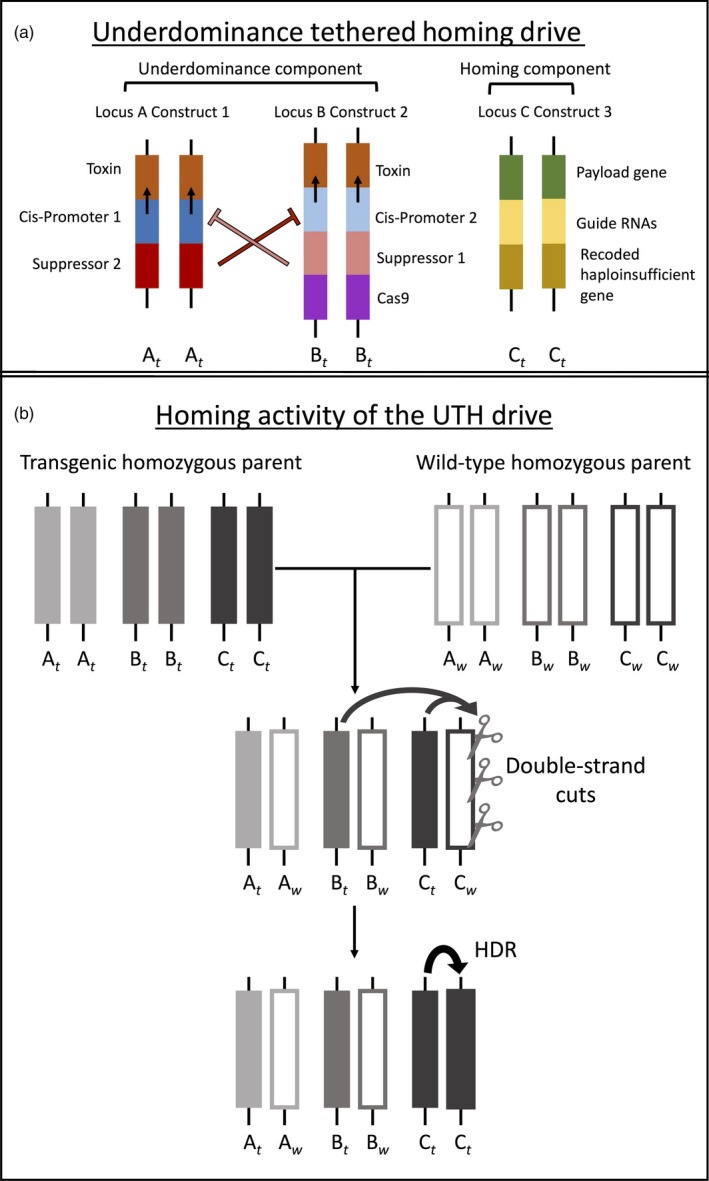
The general design and mechanism of the underdominance tethered homing (UTH) drive are shown. Different genetic elements that form a construct are shown as colored segments. (a) The three constructs of the UTH drive occupy three unlinked loci. The two underdominance constructs form a toxin‐suppressor engineered underdominance system. The third construct forms a homing component that is driven in the presence of the other two constructs. (b) In the germline of heterozygous individuals, the Cas endonuclease from the underdominance component along with the guide RNAs targets the wild‐type counterpart of the homing construct for multiple cuts. Repair through the cell's homology‐directed repair (HDR) pathway leads to insertion of the homing construct into the homologous chromosome, rendering the cell homozygous for the homing construct

The two‐locus underdominance component is structured following the design proposed by Davis et al. (2001), with the addition of sequence for germline‐specific production of a Cas endonuclease. It is composed of two constructs (Figure 1a), located at separate loci. In our model, the two loci are termed A and B, with the transgenic alleles labeled A_t_ and B_t_. The corresponding wild‐type alleles at the loci are referred to as A_w_ and B_w_. Each underdominance construct is engineered to produce a lethal toxin during early life stages, unless the individual also possesses a copy of the other underdominance construct, which harbors a suppressor for transcription of the toxin gene on the first construct (Figure 1a). Thus, only wild‐type individuals and those that carry at least one copy of each transgenic allele (A_t_ and B_t_) are viable.

Only engineered individuals that carry both underdominance constructs are viable; therefore, the sequence for the Cas endonuclease does not need to be included in both underdominance constructs. In the model shown here, it is included only in allele B_t_. Off‐target nuclease activity of Cas in the absence of any guide RNAs or even the resource cost of producing the Cas protein may impart some fitness cost to the underdominance component. We therefore assume that the allele B_t_ incurs a multiplicative fitness cost. The parameter *s*_c_ gives the cost paid by individuals homozygous for the B_t_ allele. Results with equal cost for both underdominance constructs are included in the Supporting Information (Figure S1) and are qualitatively similar to the results shown here.

The homing component of the UTH drive is located at a third, unlinked locus “C.” This component is specifically designed to target and be inserted into a haploinsufficient gene, that is, two copies of a functional gene are required at this locus for embryonic development or for gametogenesis. Engineered and wild‐type alleles at this locus are denoted C_t_ and C_w_, respectively. The homing component is composed of three tightly linked segments: (a) sequences for multiple guide RNAs that target the wild‐type haploinsufficient gene (C_w_), (b) a modified copy of the wild‐type gene that cannot be targeted by the guide RNAs, and (c) a payload gene with a promoter suited for the payload's function (Figure 1a). The homing construct is assumed to incur a multiplicative fitness cost (due to the payload and guide RNA production), where the cost paid by C_t_ homozygotes is given by parameter *s*_p_. If a TH drive is used for population suppression, a separate payload gene may not be needed when the homing construct targets a gene to specifically suppress female fitness (Kyrou et al., 2018).

In heterozygous germline cells, the guide RNAs and the Cas endonuclease together target the wild‐type (C_w_) alleles for multiple double‐stranded breaks (Figure 1b). If the damage is repaired through fully successful HDR, this results in germline cells that are homozygous for the homing construct. Repair through nonhomologous end‐joining (NHEJ) would be expected to result in a deletion at this locus due to the multiple breaks. Nonhomologous end‐joining or HDR that did not produce a functional copy of the haploinsufficient gene would be expected to result in individuals that are incapable of producing viable offspring (due to the haploinsufficiency at the locus). These two design elements, multiplexed guide RNAs and a haploinsufficient target gene, are expected to prevent the emergence of alleles resistant to the drive (Esvelt et al., 2014; Noble et al., 2016; Noble, Olejarz, Esvelt, Church, & Nowak, 2017). In a recent study in *D. melanogaster*, Oberhofer, Ivy, and Hay (2018) showed that multiplexing of guide RNAs can prevent resistant allele formation in a laboratory population. Kyrou et al. (2018) have also demonstrated that resistance to Cas9 activity can be avoided by targeting a highly conserved gene. While resistance to Cas9 targeting could still be a problem in large field populations, here we assume no resistance alleles in our model and will examine this issue in a more detailed model in the future. The homing efficiency (*H*) of the drive describes the likelihood of successful HDR at all sites identified for cutting by the guide RNAs. Thus, after the action of Cas endonuclease and the guide RNAs, an individual's fitness is reduced, on average, by a factor of (1 − *H*) due to inefficient homing. This cost represents failed gametogenesis due to failure to restore two functional copies of the haploinsufficient gene. This reduction in fitness is in addition to fitness reduction due to the costs of the two drive components (*s*_c_ and *s*_p_). Homing efficiency for CRISPR‐based drives varies widely across species and genetic constructs, but efficiencies as high as 99% have been reported (Gantz et al., 2015; Hammond et al., 2016). A recent study of a split drive, with the Cas9 and guide RNAs separated on different chromosomes, found that such separation can give comparable (and in some cases higher) homing efficiency than comparable standard homing drives (Champer et al., 2019). For the results shown in the main text of this manuscript, we use a high homing efficiency of 95% and include results with lower efficiencies in the Supporting Information. The Supporting Information also includes the relative fitness of all genotypes (Table S1) and equations for changing genotypic frequencies. Note that if haploinsufficiency is exhibited at the embryonic stage, instead of during gametogenesis, failed homing would impose a lower cost of ‘(1 − *H*)/2’ on transgenics. For this manuscript, we model the more conservative case of failed gametogenesis.

### 2.2 Two‐genotype release

When the UTH drive is introduced into a population, the release group is composed of individuals of two genotypes. A majority of individuals released carry only the underdominance component (genotype A_t_A_t_B_t_B_t_C_w_C_w_), and only a small fraction of the released individuals carry the homing component that includes the payload gene (genotype A_t_A_t_B_t_B_t_C_t_C_t_). The underdominance constructs are expected to increase rapidly in frequency in a population if introduced above a threshold frequency (Davis et al., 2001; Dhole et al., 2018; Edgington & Alphey, 2017). The lower release frequency of the homing component is intended to prevent it from imposing high indirect selection (i.e., selection due to linkage disequilibrium) against the underdominance component before underdominance reaches a high frequency (see Section 3).

### 2.3 Alternative two‐stage delayed release

Instead of a single two‐genotype release, it is also possible to introduce the UTH drive with a two‐stage delayed release, where the releases of individuals of different genotypes are separated temporally. For such an introduction, only individuals carrying the underdominance component are released initially. Individuals homozygous for the whole UTH drive are then released after a delay of 10 generations. This release scheme also allows underdominance to establish in the population by delaying the burden imposed by the cost of the payload gene.

### 2.4 Simulations

We use numerical simulations of a deterministic population genetics model to describe the dynamics of the UTH gene drive in large, well‐mixed populations with nonoverlapping generations. The three loci, described in the Drive Design section above, with either a transgenic or a wild‐type allele each, give 27 diploid genotypes. Within each generation, the genotypic frequencies of the offspring born in each population are modified by fitness effects of the gene drive components to give adult genotypic frequencies. The action of the endonuclease and guide RNAs (homing; see details below) in the germline results in new genotypic frequencies in the gametes, which may be carried across populations by migrating adults. Individuals are assumed to mate randomly with respect to their genotypes at the gene drive loci after migration. Our simulations track the frequencies of these genotypes across generations after the initial release of the gene drive into the target population. We assume a 1:1 sex ratio at birth and that all drive‐based natural selection (fitness impacts of constructs, segregational cost of underdominance, and inefficient homing) occurs before mating (in pre‐adult stages). This may reduce the size of the breeding adult population within a generation, but we assume that the same number of offspring is born every generation. This assumes perfect density compensation. Note that this assumption is implicit (although not always explicitly mentioned) in all frequency‐based models of gene drive evolution where the number of individuals is not tracked and large population sizes are assumed (Altrock, Reeves, & Reed, 2010; Davis et al., 2001; Edgington & Alphey, 2017; Huang et al., 2007; Marshall & Hay, 2012b; Noble et al., 2017). We first address drive dynamics in a single population and then use a two‐population setting to study the level of localization for the UTH gene drive. Mathematica (Wolfram Research, Inc.) code for the simulations is available on DRYAD (https://doi.org/10.5061/dryad.70dn712).

Two factors that strongly influence the performance of a homing‐based gene drive are the fitness costs of the drive components (here given by parameters *s*_c_ and *s*_p_) and the homing efficiency (given by parameter *H*). The underdominance component of the UTH drive only serves the purpose of providing the Cas endonuclease for the homing construct. Therefore, the same underdominance constructs can be used for substantially different applications of the gene drive. The costs of the payload gene, on the other hand, will vary depending upon the purpose of the gene drive release. Homing efficiencies for CRISPR/Cas‐based homing drives vary across species and across genetic constructs (Champer, Liu, et al., 2018). We analyze the release effort required to alter an isolated population with a UTH drive across a range of possible values for the cost of the underdominance component, for payload costs, and for the homing efficiency.

The results shown below are from simulations of a single two‐genotype release, with the exception of two cases that are highlighted. We simulate a release where engineered individuals of both sexes are introduced. The starting frequency, after the single initial release, for the homing construct is 5%, while the frequency of the underdominance component varies from 5% to 90%. As a measure of population alteration, we use the mean frequency of the gene drive constructs over a 100‐generation period after the release. The mean frequency gives a more accurate representation of population alteration than the final allelic frequencies.

### 2.5 Capacity for population suppression

It is clear that a gene drive intended for population suppression must impose a high genetic load on the target population (i.e., reduction in the mean fitness or reproductive capacity of individuals in the altered population compared to a population composed entirely of wild‐type individuals) (Burt, 2003). Gene drives that can impose high genetic loads, for sustained periods, are likely to achieve substantial population suppression (Burt, 2003; Sinkins & Gould, 2006). A rigorous analysis of population suppression would require a model that can incorporate biological details much beyond the scope of our proof‐of‐concept model. Details of a population's density‐dependent dynamics, mating behavior, and stochasticity are expected to play major roles in determining how a population responds to genetic load imposed by any gene drive (Backus & Gross, 2016; Edgington & Alphey, 2018; Khamis et al., 2018). For many species, spatial dynamics within a population will also likely influence gene drive dynamics and the level of population suppression (Champer, Zhao, et al., 2018, see also Barton, 1979a, Barton & Rouhani, 1991). Migration between multiple populations can further complicate suppression analysis, with genetic drift and stochasticity in movement affecting the spread of genetic elements (Barton, 1979a; Marshall & Hay, 2012a). Analysis of how these different factors can influence the dynamics of TH gene drives will require multiple future models.

To gain a comparative measure of the capacity of the UTH drive for population suppression, we use the level and duration of genetic load it can impose, and compare this with similar measures for previously proposed gene drives. We show the mean genetic load imposed by the UTH drive on an isolated population after single two‐genotype releases of varying sizes. Reducing female viability (or fecundity) has a stronger influence on population genetic load than reducing male viability (or fertility). Suppression drives that only reduce female fitness are also likely to be more efficient, because natural selection acting against the drive would be weak in males. A homing drive that disrupts female fecundity was recently shown to be capable of completely eradicating laboratory populations of *Anopheles gambiae* (Kyrou et al., 2018). Also, a payload with recessive fitness costs is expected to face weaker natural selection when at low frequencies, facilitating easier initial spread (Magori & Gould, 2006). For these reasons, we include modeling of a UTH drive with a payload gene that only reduces female fitness by imposing a recessive fitness cost. Results with multiplicative fitness costs are provided in the Supporting Information (Figure S2).

### 2.6 Gradually increasing the genetic load

For certain applications, it may be desirable to be able to gradually increase or adjust the genetic load in a population. For instance, when the desired outcome is population suppression without complete eradication, gradually increasing the genetic load can help avoid density‐dependent effects, such as Allee effects or drift, from driving the population extinct, which would leave the habitat open to recolonization by immigrants. A useful feature of the UTH drive is that it can be used to successively drive multiple payload genes into a population with minimal release effort after the initial establishment of the drive. The additional payloads would be released as individuals with separate homing constructs (transgenic alleles at new loci D, E, and so on) linked with new guide RNAs that would use the pre‐established Cas gene. We simulate the spread of three payload genes designed for reducing female fitness, successively released at 20‐generation intervals.

### 2.7 Migration and localization analysis

To assess the level of localization of the UTH drive, we use a scenario with two populations, a target and a neighbor population, that exchange migrants every generation. We assume that the two populations are initially of equal size. However, the spread of the gene drive may alter population size within a generation if the gene drive constructs are costly. We assume that each adult individual has a fixed probability of migrating out of its native population before mating. Thus, a constant fraction, *µ*, of individuals from each population leaves to join the other population. This means that if the number of adults in one of the population becomes smaller than the other, a smaller absolute number of individuals migrate out of it, relative to those migrating out of the larger population (Dhole et al., 2018). This is more realistic than fixed migration rates that imply the same number of individuals migrate irrespective of a population's size. We term the effective immigration rate into the target population as *µ*_T_ and that into the neighbor population as *µ*_N_. We account for the potential differences in effective immigration rates (due to differences that may arise in adult population size) with the approximations.

(1) $$\begin{array}{c} \mu_{\text{T}}=\mu P\\ \mu_{\text{N}}=\frac{\mu }{P} \end{array}$$

where *P* is a proxy for the ratio of the population sizes, given by

$$P=\frac{\left(1-L_{T}\right)}{\left(1-L_{N}\right)}$$

here, *L*_T_ and *L*_N_ are the genetic loads in a given generation in the target and the neighboring populations, respectively. Equation 1 allow for an asymmetry to arise in the effective immigration rates if the two populations begin to differ in size (when *P* ≠ 1). If both populations are of equal size (when *P* = 1), migration would be symmetrical. Note that Equation 1 do not incorporate changes in population size over multiple generations, but only within a generation (reduction from offspring to adult stage). Explicitly modeling across‐generation changes in population size would require a model that can incorporate population density and spatial dynamics, which are beyond the scope of this model (but see Barton, 1979a,1979b; Barton & Rouhani, 1991; Lande, 1985 for analyses of density effects on the spread of threshold‐dependent genetic elements). Analyses without any correction for differences in population size are included in the Supporting Information: Section 6

We follow the frequencies of the three gene drive constructs (the three transgenic alleles at the three loci) in both populations through 100 generations after the initial release. Localized spread of the gene drive would result in successful alteration of the target population, while leaving the neighbor population largely unaltered.

## 3 RESULTS

### 3.1 Dynamics in an isolated population

**Figure 2:**
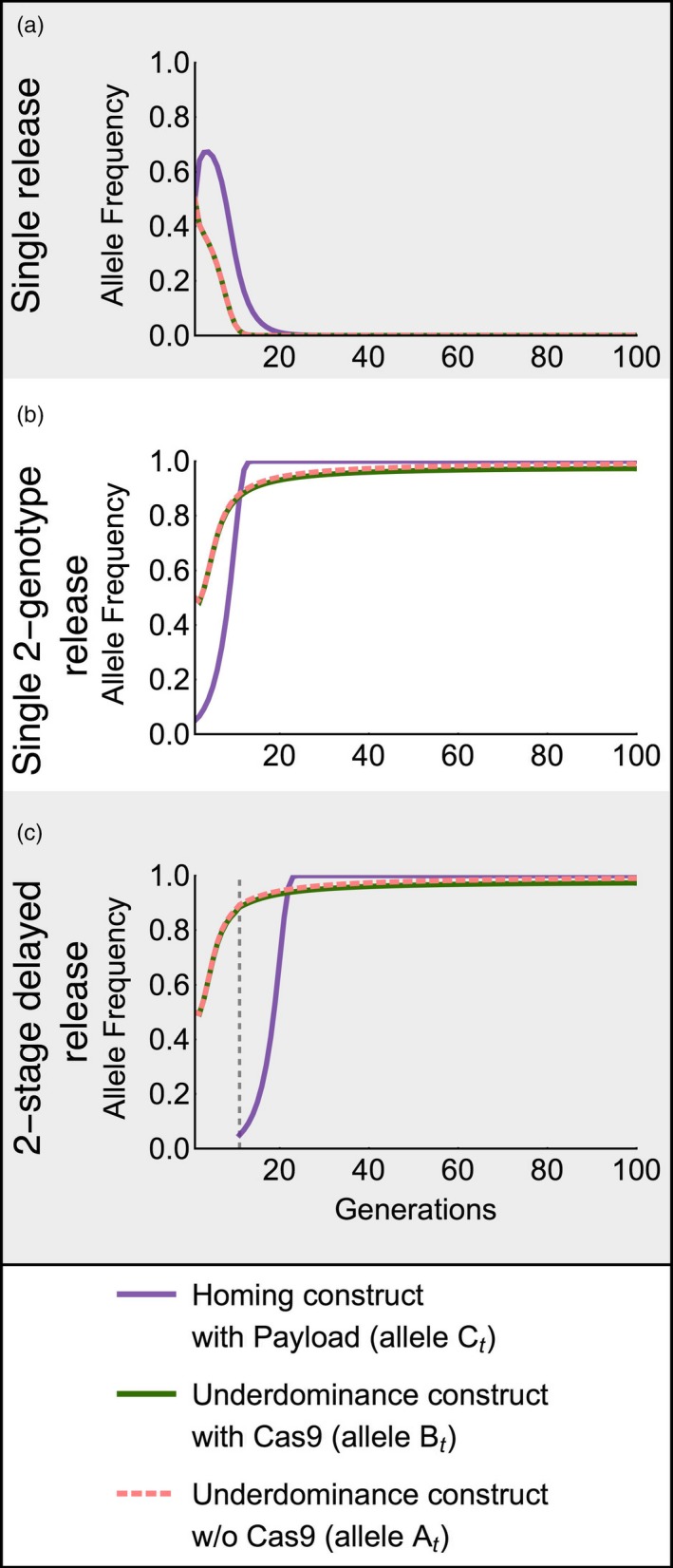
Time‐series of different release methods for the UTH drive is shown, after a release of engineered individuals at a 1:1 ratio to wild‐type individuals. Homing efficiency, *H* = 0.95; underdominance construct cost for B_t_B_t_ homozygotes, *s*_c_ = 0.05; homozygous payload cost, *s*_p_ = 0.5

The two‐genotype release is a critical feature needed for efficient use of the UTH drive. Introducing the UTH drive into a population as a one‐genotype release of individuals homozygous for the complete drive (genotype A_t_A_t_B_t_B_t_C_t_C_t_) results in drive failure, unless the release is extremely large or all drive constructs, including the payload, have extremely low fitness costs. For instance, a UTH drive carrying a payload with a 50% homozygous fitness cost fails after a single one‐genotype introduction at 1:1 release ratio with wild‐type (Figure 2a). However, a two‐genotype release of the same size (1:1 release giving a starting frequency of 0.5 for alleles A_t_ and B_t_, and 0.05 for allele C_t_) results in successful drive (Figure 2b). The reason for the major difference between the success of these release approaches is that the cost of the homing construct (which carries the payload) results in strong selection against all components of the UTH drive, because strong linkage disequilibrium develops between the three loci upon release. The low initial frequency of the homing construct in a two‐genotype release lets underdominance reach a high frequency, which then successfully drives the homing component to a high frequency. A two‐stage delayed release of the homing component can similarly allow successful drive (Figure 2c).

**Figure 3:**
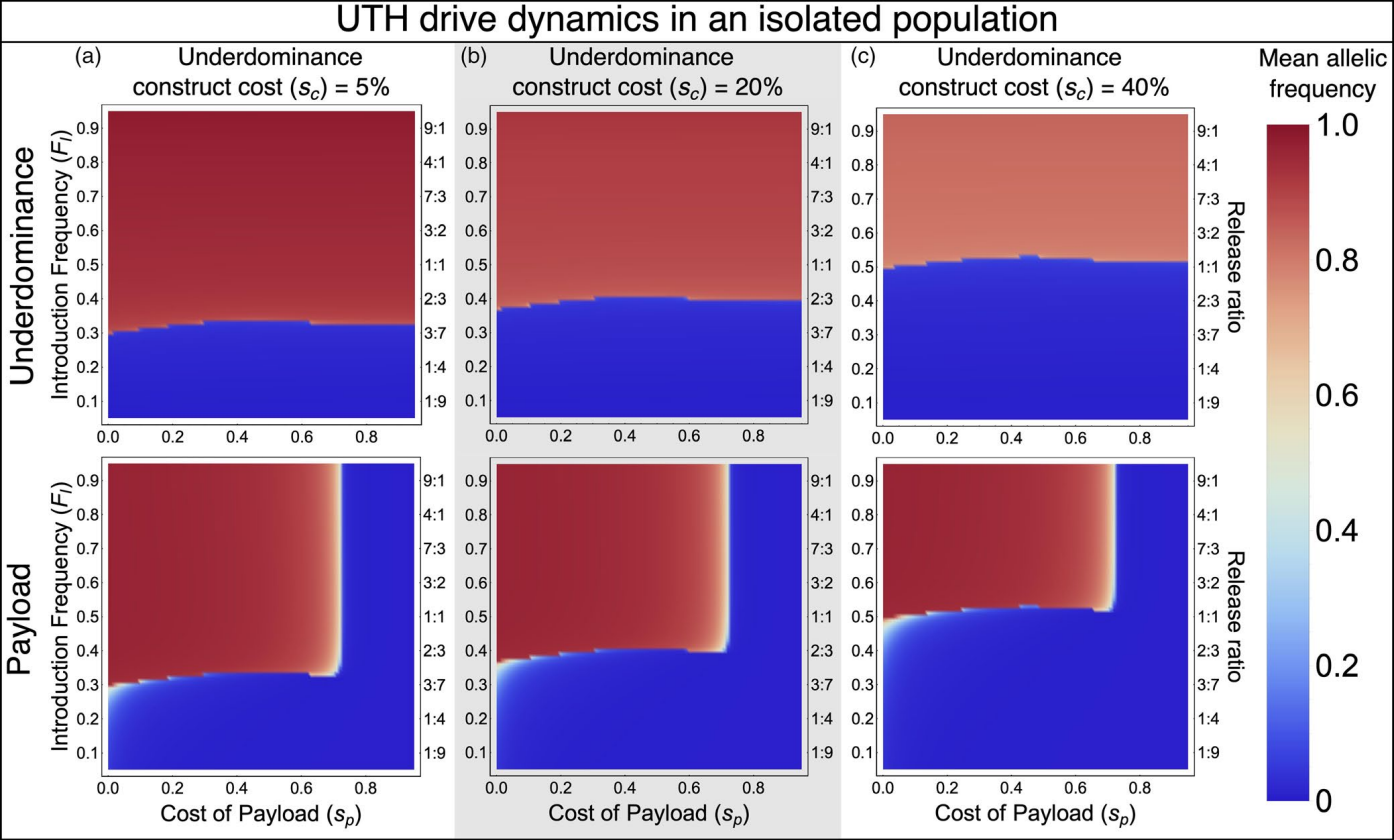
Underdominance tethered homing (UTH) drive in an isolated population—colors show mean allelic frequencies for one of the underdominance (allele B_t_, top row) and the payload gene (bottom row) over a 100‐generation time span following a single release. The three columns show results for UTH drives with different fitness costs of the underdominance component

The underdominance component causes the UTH drive to have a threshold release frequency that needs to be exceeded for successful population alteration. As expected for any case of underdominance (Altrock et al., 2010; Barton, 1979b; Bazykin 1969; Barton & Turelli, 2011; Edgington & Alphey, 2017; Magori & Gould, 2006; Reeves et al., 2014; Sinkins & Gould, 2006), higher costs of the underdominance component result in higher threshold frequencies for the UTH drive (Figure 3 top row). Even when the release exceeds the threshold, higher costs of the underdominance component lower the equilibrium frequency of the underdominance constructs of the UTH drive in the population as indicated by the lighter red color representing the mean frequency over 100 generations. However, note that even these lower equilibrium frequencies of the underdominance constructs are sufficient to successfully drive the payload gene on the homing construct to high frequencies (Figure 3 bottom row). When the drive is released below the threshold, population alteration fails completely as none of the drive components spread (indicated by the dark blue color in the top and bottom rows of Figure 3). When the payload cost is too high, the drive fails as the homing construct is lost and only the underdominance component becomes established in the population (Figure 3; red area in top panel with corresponding blue area in bottom panel).

The ability of the UTH drive to push a payload gene into an isolated population can be compared with that of three other gene drives for which we have previously performed identical analyses: one‐ and two‐locus engineered underdominance drives and the daisy‐chain drive (Dhole et al., 2018). Mean payload frequencies achieved with the UTH drive when assuming a 5% fitness cost of the underdominance (bottom panel of Figure 3a) can be compared to figure 3 of Dhole et al. (2018). In the simple engineered underdominance drives analyzed in Dhole et al. (2018), the payload is directly linked to one of the underdominance constructs. This comparison shows that when the payload has high fitness costs, the UTH drive is dramatically more efficient than the simple engineered underdominance drives. The UTH drive can drive a payload gene with a given cost to a much higher frequency and with smaller release that can be done with a simple engineered underdominance drive. The daisy‐chain drive requires the lowest release, but has other drawbacks compared to the UTH drive (see results on localization in Dhole et al., 2018).

Results shown in Figure 3 are for simulations with 95% homing efficiency and equal payload fitness costs to both sexes. Lower homing efficiencies restrict the maximum payload gene cost that can still allow successful population alteration. However, even with homing efficiencies as low as 70%, the UTH drive can successfully spread payloads with almost 50% homozygous fitness cost to both sexes, although the gene drive requires a large number of generations for complete population alteration (see Figure S3).

### 3.2 Capacity for population suppression

**Figure 4:**
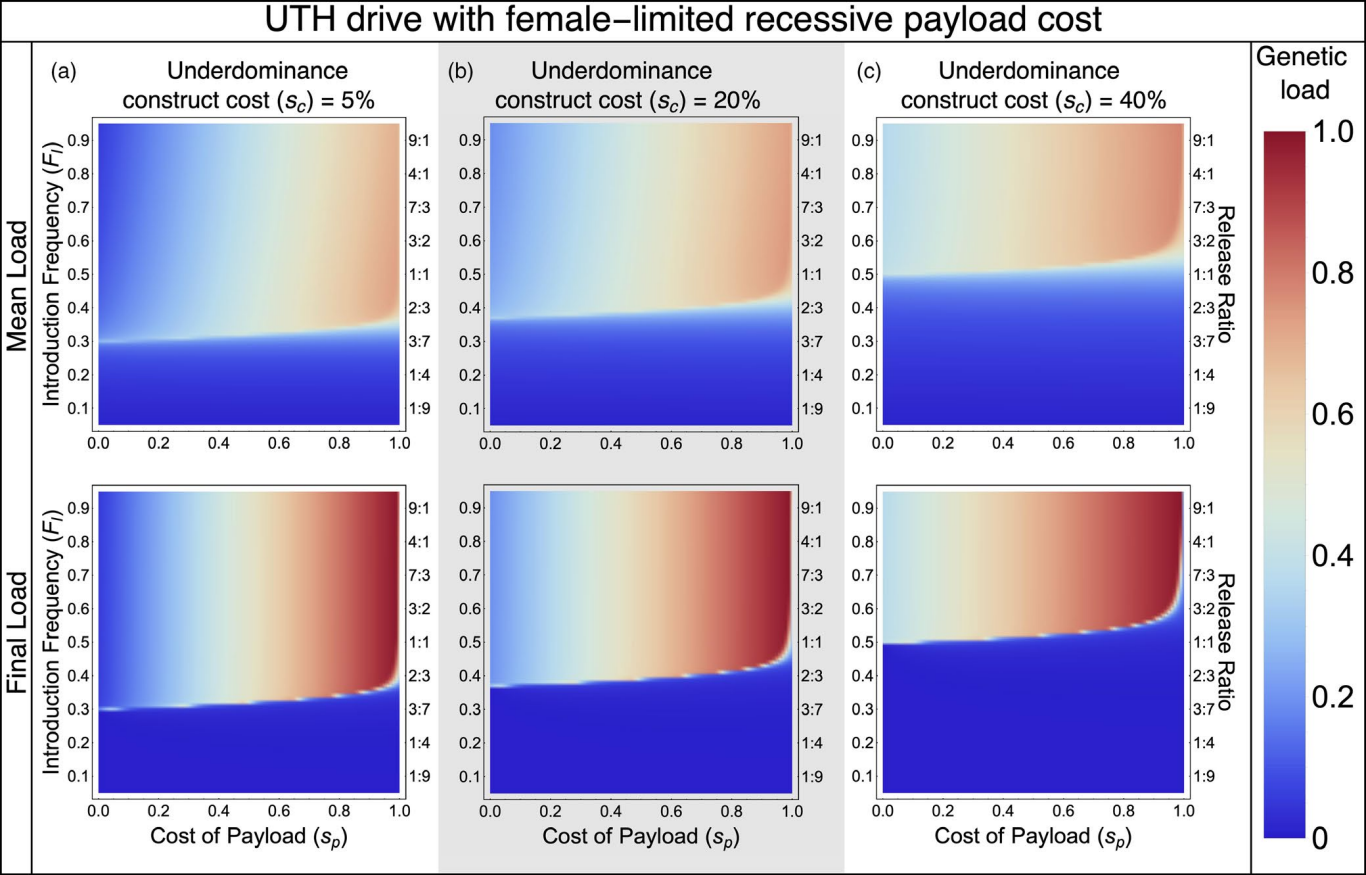
Colors show mean genetic load over twenty generations after drive release (top row) and final genetic load in the twentieth generation (bottom row)

A UTH drive designed for population suppression through high female‐limited fitness costs can impose a considerable genetic load on a population (Figure 4). The underdominance component of the UTH drive imparts some genetic load due to the direct fitness cost of the constructs (*s*_c_) and the segregational cost of underdominance itself (lower fitness of heterozygotes). However, a large fraction of the genetic load is accrued through the spread of a costly payload located on the homing construct (*s*_p_). As the homing construct of the UTH is released at a low frequency, the genetic load builds up slowly. The mean genetic load in the first 20 generations after releasing the drive is therefore lower than the genetic load imposed in the 20th generation—“final load” (Figure 4). For example, even with a very large release, the highest mean genetic load in the first twenty generations remains below 0.8, while the genetic load in the 20th generation can reach very close to unity for very costly payloads.

**Figure 5:**
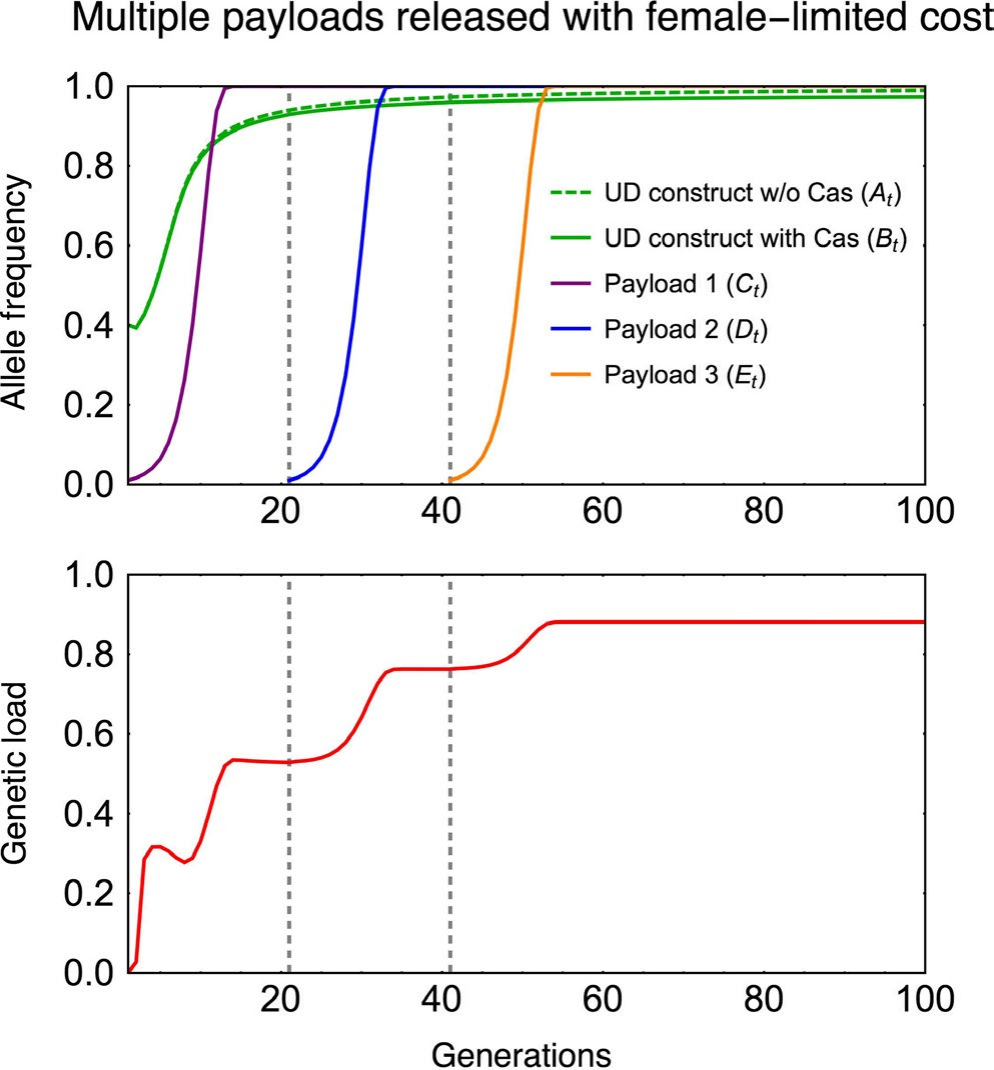
Time‐series plots show frequencies of successively released homing constructs with new payloads (top panel) and the gradual buildup of genetic load in the population with the spread of each new payload gene. The first release has starting frequency of underdominance component at 40% and the first payload at 1%. Each successive payload is released to achieve 1% starting frequency. Each payload gene has a 50% female‐limited homozygous fitness cost. Dashed gray lines show the time of release of successive payloads. Other parameters are *s*_c_ = 0.05, *H* = 0.95

**Figure 6:**
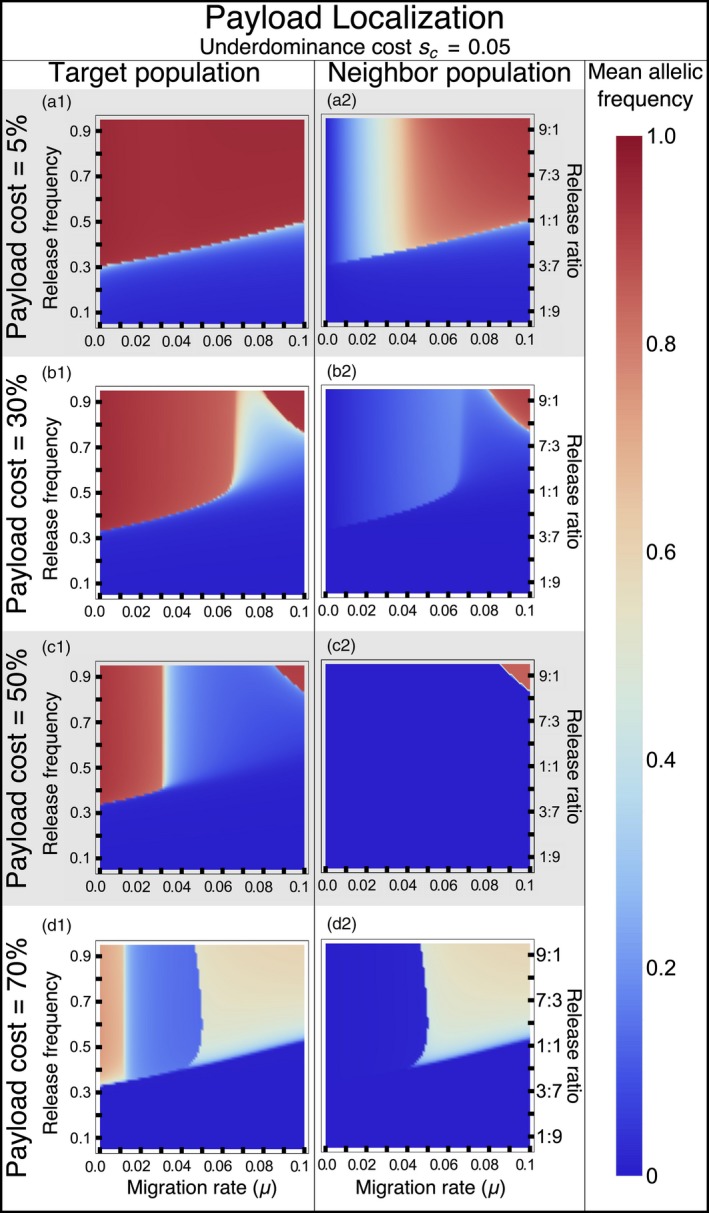
Localization of the UTH drive: Colors show mean frequency of the payload allele over 100 generations in the target and a neighbor population. Cost of the underdominance component, *s*_c_ = 0.05. Different rows show UTH drives with different homozygous payload costs. The two‐genotype drive is released to attain a starting homing construct frequency of 0.05. The starting frequency of all released individuals (including those with the full drive complement and those with only the underdominance component) is given on the vertical axis in each panel. Payload costs described are for homozygotes of both sexes

With successive release of multiple payloads on different homing components, the UTH drive can be used to gradually adjust (increase or decrease) the genetic load imposed on a population. For example, successive release of three payload genes with female‐limited fitness reduction can be used to gradually increase the genetic load on a population to a high level (Figure 5). Conversely, a “rescue” payload gene can be driven to reduce the genetic load imposed by a loss of function payload gene. The total number of transgenic individuals released to spread the three payloads is only slightly higher than that for spreading a single payload, because after the initial establishment of the underdominance constructs, each additional payload only needs to be released at very low frequency (1% for the example shown in Figure 5). The UTH drive can achieve high genetic loads using a broader set of parameters than with the other drives examined in Dhole et al. (2018—see figure 6 and figure S11 therein).

When payload genes affect fitness of both sexes, sequentially driving multiple payload genes that each have a small effect on fitness can achieve a much higher combined genetic load compared to what can be achieved by driving a single high‐cost payload that affects both sexes (Figure S4).

### 3.3 Localization of the gene drive

**Figure 7:**
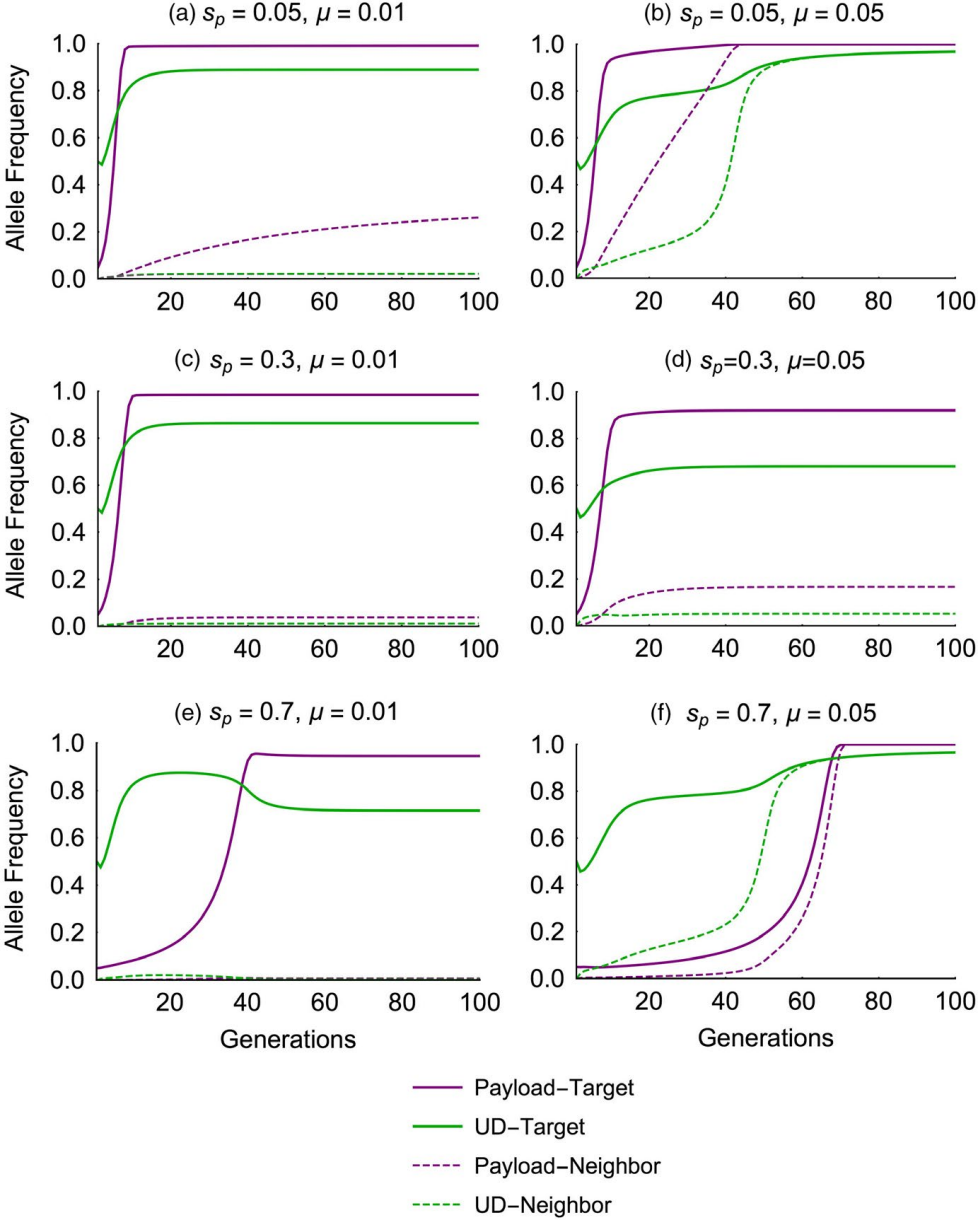
Time‐series plots show the allelic frequencies of the payload (allele C_t_, purple lines) and the underdominance with Cas endonuclease (allele B_t_, green lines) in the target (solid lines) and the neighboring (dashed lines) populations. Individual plots show dynamics with low (*µ* = 0.01, left column) or high (*µ* = 0.05, right column) migration rates, and with different payload costs (across rows). Dynamics of the underdominance construct without Cas (allele A_t_) are similar to that of allele B_t_ and are not shown for visual clarity

Similar to other genetic elements that exhibit threshold‐dependent spread (i.e., exhibit bi‐stable dynamics; Barton, 1979b; Barton & Turelli, 2011; Lande, 1985; Marshall & Hay, 2012a), the influx of wild‐type individuals into the target population through migration increases the threshold frequency for the UTH drive (Figure 6). The level of localization of the drive depends upon the fitness costs associated with its two components. A UTH drive with a low‐cost underdominance component (*s*_c_ = 0.05) fails to remain localized when driving a low‐cost payload gene designed for population replacement (*s*_p_ = 0.05), unless migration rates are <1% (Figures 6a and 7a,b). However, the UTH drive can achieve localized population alteration with high‐cost payload genes over a much broader range of migration rates compared to simple one‐locus engineered underdominance drive or the daisy‐chain drive (Figures 6b–d and 6, 7c–e).

A UTH drive created with a low‐cost underdominance component is not more localized than a simple two‐locus underdominance drive. However, a few modification to the UTH drive shown in Figure 6 can greatly improve its localization. For example, a UTH drive with an underdominance component with slightly higher fitness cost (*s*_c_ = 0.2) has localization level similar to that of a simple two‐locus engineered underdominance drive when driving high‐cost payload genes (Figure S6; see Dhole et al., 2018). Moreover, a modified, though perhaps more complex, design for a UTH drive can be much more localized than a two‐locus underdominance drive. The modified design includes a third copy of the toxin gene on the homing construct that is suppressed by one of the suppressors on the underdominance component (Figure S7). The toxin gene on the homing construct from this modified UTH drive prevents the payload gene diffusing into a neighboring population without the underdominance constructs (Figure S8).

For a small range of extremely high payload costs (Figures 6d and 7e,f), a UTH drive with low‐cost underdominance component can actually become less localized than a drive when payload costs are low. This is because the extremely high payload costs keep the frequency of the homing construct (which carries the payload) at low levels until underdominance (which here confers only 5% fitness cost) becomes established in both populations, subsequently allowing the payload to spread in both populations (Figure 7f). As described earlier for result shown in Figure 2, linkage disequilibrium with a costly payload gene results in selection against the underdominance component. When migration is high and the payload is extremely costly (Figure 7e), underdominance components can become established in the neighboring population without becoming encumbered by the payload, because payload frequency in the neighbor population is very low (allowing little linkage disequilibrium). In the case when the payload is not very costly (Figure 7b), underdominance components are similarly unencumbered because payload is fixed in both populations (again allowing little linkage disequilibrium). Note that in the case of low‐cost payload (Figure 7b), even if higher linkage disequilibrium existed, indirect selection against the underdominance component would be weak due to the low cost of the payload gene. This spread of the underdominance component to the neighboring population with high‐cost payload does not occur with the modified versions of the UTH drive with higher cost for the underdominance component itself (Figures S6 and S8).

## 4 DISCUSSION

Recently, spatially and temporally restricted gene drives have gained considerable attention as having less risk compared to unrestricted drives (NASEM, 2016). The focus of most of the concern has been related to the risk of unrestricted gene drives aimed at population suppression or eradication. A number of strategies for spatially restricted gene drives have been outlined (Akbari et al., 2014; Buchman et al., 2018; Burt & Deredec, 2018; Davis et al., 2001; Marshall & Hay, 2012a; Noble et al., 2016; Oberhofer et al., 2019; Rasgon, 2009) and a few have been tested on *Drosophila* in the laboratory (Akbari et al., 2014, 2013; Buchman et al., 2018; Oberhofer et al., 2019; Reeves et al., 2014), but mathematical models indicate that while these approaches are reasonable for changing characteristics of local populations (i.e., population replacement), they are less likely to be effective in suppressing local populations that have realistic density‐dependent dynamics (but see Khamis et al., 2018; Marshall & Hay, 2014).

In general, it is difficult to effectively add high‐cost payloads to gene drives that intrinsically have high release thresholds (Dhole et al., 2018; Marshall & Hay, 2012a). Unfortunately, gene drives with low intrinsic thresholds are less likely to remain localized. The work outlined here was aimed at developing an approach that would enable addition of substantial fitness cost to one spatially restricted drive strategy, two‐locus underdominance, while maintaining an attainable release threshold. The localization level of a TH drive is highly dependent on the drive used as the anchor. The dynamics shown here are specific to a TH system based on two‐locus engineered underdominance. A benefit of the TH system is that it can easily be implemented with any localized gene drive that can be linked with sequence for producing Cas. Tethering a homing construct with a localized gene drive system essentially adds stronger driving capability to the localized drive (in terms of driving costlier payloads) while still maintaining comparable localization level.

Our general proof‐of‐concept model ignores many biological factors that can influence the spread and localization of gene drives in real populations (Altrock et al., 2010; Barton, 1979b; Barton & Turelli, 2011; Champer, Zhao, et al., 2018; Edgington & Alphey, 2017; Huang, Lloyd, Legros, & Gould, 2011). For instance, in finite populations, factors such as genetic drift and density gradients can have a large influence on the spread of genetic elements that exhibit a release threshold (including all spatially restricted gene drives), especially when movement across populations is stochastic (Barton & Rouhani, 1991; Barton & Turelli, 2011; Lande, 1985; Marshall & Hay, 2012a). Stochasticity in movement between populations can result in episodes of high migration even when average migration rates are low. Such episodes can allow a threshold‐based gene drive to invade neighboring populations more easily, and it can also impede the spread of the gene drive in the target population with a large influx of wild‐type immigrants. Density‐dependent dynamics will also influence the spread of gene drives that impose high genetic loads as it would alter the number of transgenic versus wild‐type individuals that move across population boundaries (Barton, 1979b; Champer, Zhao, et al., 2018; Sudweeks et al., 2019). Differences between populations in size and density also play an important role (Barton, 1979a,1979b; Barton & Rouhani, 1991; Barton & Turelli, 2011; Lande, 1985). The equations for effective immigration rates in our model allow asymmetric migration to occur when a population becomes smaller due to suppression. Although our simulations take into consideration the effects of genetic load in the target population on the rate of migration, our model does not address the scenario where the target population is initially much larger or denser than the neighboring population. Such a scenario may be especially relevant to gene drive applications, as pest management is likely focus on the more dense populations. Also, the release of transgenic insects would initially raise the density in the target population. A scenario where the target population is much larger than the neighboring population can result in a large number of transgenics migrating into the neighboring population, reducing the level of localization for a TH drive (Barton, 1979a,1979b).

The rich body of previous theoretical work that has been done on the spread of bi‐stable genetic elements (Altrock et al., 2010; Barton, 1979b; Barton & Hewitt, 1985; Barton & Rouhani, 1991; Barton & Turelli, 2011; Lande, 1985; Michalakis & Olivieri, 1993; Pialek & Barton, 1997; Turelli & Hoffmann, 1995) is useful for making some of these qualitative predictions regarding how the multitude of biological factors would influence the dynamics of a tethered homing drive. However, a significant difference between tethered homing drives and the bi‐stable elements previously considered is that only one of the two components of a TH drive exhibits bi‐stability (i.e., exhibits threshold behavior)—the anchor drive (the two‐locus engineered underdominance in a UTH drive). The dynamics of the homing component are Fisherian (i.e., do not show a threshold‐dependent behavior). Also, much of the previous theoretical work on natural bi‐stable elements assumes weak selection, which is not the case for gene drives that are used for population suppression. Thus, a complete, quantitative treatment of the dynamics of this new gene drive in natural populations would require multiple future studies. But the outcomes from this model are encouraging, especially for cases of invasive populations on oceanic islands where migration rates from the target population to a neighboring population are low. Use of gene drives for aiding conservation efforts has been discussed for cases such as invasive rodents that have invaded oceanic islands and are contributing to extinction of native flora and fauna (Bellard, Cassey, & Blackburn, 2016), or the mosquito, *Culex quinquefasciatus*, that transmits bird malaria to the endangered Hawaiian honeycreepers (Atkinson, Woods, Dusek, Sileo, & Iko, 1995; van Riper, Riper, Goff, & Laird, 1986; Woodworth et al., 2005). For such cases, any potential use of gene drives for population suppression would require a localized approach that can impose high genetic load. In the case of *Culex quinquefasciatus*, a localized gene drive that alters the mosquito population so that it cannot transmit the malaria pathogen could also be effective. The TH drive could be useful for either approach.

For payloads with equal impacts on male and female fitness, a UTH drive can impose much higher genetic load than a two‐locus underdominance drive, and the release thresholds are substantially lower. For example, a UTH drive can establish a payload with 50% fitness cost with a much smaller release and under much higher migration rates compared to a two‐locus underdominance drive (compare Figure 6c here with figure 5h in Dhole et al., 2018). If the payload only has an impact on female fitness (Figure 4), then the genetic load from the UTH on a local population can reach above 0.9 within 20 generations (i.e., only a few years for mice and mosquitoes on tropical islands).

As we mention above, density dependence, as well as other factors such as mating behavior, stochasticity, spatial dynamics, can give rise to complex population dynamics in response to perturbations. Therefore, the relationship between a specific level of genetic load and the impact it has on population density or persistence is not straightforward. Recent analyses (Backus & Gross, 2016; Edgington & Alphey, 2018; Khamis et al., 2018) also highlight that the specific values of several ecological parameters can have a large influence on the success of a gene drive. One advantage of the UTH system is that once one payload is established in a population, it is possible to add successive payloads (Figure 5) by releasing a small number of additional individuals. One could start with a payload that results in a relatively low genetic load and that load could be increased as needed. It is important to recognize that a genetic load that decreases the density of an invasive pest but does not cause eradication could be more sustainable than a high load that causes local extinction. A drive with moderate load would remain active in the target population and would therefore impact any rare immigrants arriving from other populations. The ability to adjust the genetic load would be helpful in these cases where the goal was just to lower the population density below a harmful level. In cases where payload transgenes with female‐limited fitness effects are not easily available, the ability of UTH drive to successively drive multiple payloads can be used to achieve higher genetic loads than are possible with single payload genes that affect both sexes (Figure S4).

The level of localization of a UTH drive could be improved by designing the underdominance component to have a higher fitness cost (Figure S6), or by including a copy of the toxin gene on the homing construct that is suppressed in the presence of the underdominance constructs (Figure S7). This third copy of the toxin gene would impose a genetic background‐dependent cost on the homing construct, so that it can spread in the target population largely unimpeded by the toxin, but be eliminated quickly from the neighboring populations that have very low levels of the underdominance constructs (Figure S8).

The feasibility of these systems as useable tools would depend on the practical difficulties involved in engineering these components as well as their effects on fitness in natural conditions. Developing toxin–antidote genetic constructs remains a difficult task, but the recent development of a toxin–antidote system in a mosquito (S. Webster‐Tostenson, personal communication) is an encouraging step forward. It should also become feasible in the near future to develop underdominance systems that are based on Cas proteins that alter gene expression instead of exhibiting endonuclease activity (reviewed in Kampmann, 2018; Knott & Doudna, 2018). For instance, two constructs, each with an RNA‐binding Cas13b, guide RNAs, and a toxin‐coding gene, can be used to create a two‐locus underdominance system—The Cas13b on a construct is guided to target the mRNA products of the toxin‐coding gene from the other construct. Of course, the feasibility of using Cas‐based underdominance for creating tethered homing drives will require prevention of cross‐targeting by the two types of Cas molecules from the underdominance and homing constructs.

Although we use engineered underdominance as an example anchor in our model, the TH approach can be implemented with any other localized system currently under development, such as chromosomal translocations. An intriguing possibility is offered by the recent publication by Oberhofer et al. (2019) that outlines the Cleave and Rescue (CleaveR) gene drive concept and demonstrates its potential in a *Drosophila* system. The CleaveR gene drive is composed of a Cas9‐guide RNA construct (“cleaver”) that targets and destroys an essential gene, and that is linked to a modified copy of the essential gene (“rescue”) that cannot be targeted by the guide RNAs (Oberhofer et al., 2019). When the essential gene targeted by CleaveR is haploinsufficient, the drive behaves like an underdominance drive with a release threshold (Oberhofer et al., 2019). The construct created by Oberhofer and colleagues targets a gene that is haplosufficient (at least in a laboratory environment) and therefore has a lower release threshold than that expected for a CleaveR system targeting a haploinsufficient gene. We propose that this system can be used to create a tethered homing drive with few modifications. As the CleaveR construct already contains a Cas9 assembly with germline expression, the only modification required for creating a TH drive is the addition of a homing construct (Figure S9). The level of localization of a CleaveR‐based TH drive would be even higher if the construct created by Oberhofer and colleagues can be modified to target a haploinsufficient gene (Figure S9). With the robust functionality of the CleaveR system (Oberhofer et al., 2019), these modifications are likely to be much more feasible than constructing a novel toxin–antidote system.

Given the various limitations of localized gene drive systems, safe alteration of natural populations may require combining multiple approaches, with potential redundancies in mechanisms that improve localization. For example, a TH drive with a homing construct that targets a private or locally fixed wild‐type allele (Sudweeks et al., 2019) is likely to be much more localized than a TH drive that targets alleles present at high levels in multiple populations. The localization of a gene drive designed for population suppression could also be improved if the payload gene that reduces fitness also reduces the likelihood of migration of transgenic individuals, for example, by reducing mobility or tolerance to the elements during migration.

All of the results presented here come from a general model designed to introduce the concept of tethered homing drives and should not be overinterpreted as predicting an outcome in a specific case or immediate suitability for use in real populations. The analyses shown here are intended to facilitate a comparison between the general behaviors of the UTH drive with previously proposed gene drives. More detailed models that reflect the biology of a targeted population of a species will be needed for assessing whether the UTH drive or related TH drives are appropriate for a given problem. But the present results suggest that tethered homing drives could prove a useful tool for localized population alteration for conservation or epidemiological purposes.

## CONFLICT OF INTEREST

None declared.

## Data Availability

Mathematica code for the mode and all figures is deposited in DRYAD (https://doi.org/10.5061/dryad.70dn712; Dhole, Lloyd, & Gould, 2019).
