# Supplementary material for: Tethered homing gene drives: A new design for spatially restricted population replacement and suppression
Source: Evol Appl. 2019 Jun 17;12(8):1688–702. doi: 10.1111/eva.12827 (PMC6708424; doi:10.1111/eva.12827)
Supplement: Supplementary file 1 [file EVA-12-1688-s001.docx]

**Supplementary material**

**Section 1: Relative fitnesses of genotypes and recursion equations for genotypic frequencies**

The cost of carrying the transgenic constructs, the mortality due to toxin production (when it is not suppressed) and the effect of gamete loss due to inefficient homing reduce the relative fitness of individuals bearing transgenic alleles. Table S1 below describes the relative fitnesses of the different genotypes formed by the haplotypes shown in row and column headings. Shown here are the effect of multiplicative fitness costs of the constructs, which are used for most of the results shown in the main text.

**Table S1 Relative genotypic fitnesses:** Average relative fitness of individuals is shown, with different columns and rows showing parental haplotypes, where wildtype individuals have a fitness one. The underdominance construct carrying the Cas endonuclease (allele B*_t_*) incurs a multiplicative fitness cost given by parameter *s_c_*. Thus each B*_t_* allele reduces individual fitness by a factor (1-*s_c_*)^1/2^. Similarly, the homing construct carrying the payload gene reduces a fitness due to multiplicative cost given by *s_p_*. Failed homing in C*_w_*C*_t_* individuals that also carry a B*_t_* allele reduces their fitness by factor of *H*, which gives the homing efficiency.

|  | **A*_w_*B*_w_*C*_w_*** | **A*_w_*B*_w_*C*_t_*** | **A*_w_*B*_t_*C*_w_*** | **A*_w_*B*_t_*C*_t_*** | **A*_t_*B*_w_*C*_w_*** | **A*_t_*B*_w_*C*_t_*** | **A*_t_*B*_t_*C*_w_*** | **A*_t_*B*_t_*C*_t_*** |
| --- | --- | --- | --- | --- | --- | --- | --- | --- |
| **A*_w_*B*_w_*C*_w_*** | 1 | (1-s*_p_*)^1/2^ | 0 | 0 | 0 | 0 | (1-*s_c_*)^1/2^ | (1-*s_c_*)^1/2^(1-*s_p_*)^1/2^*H* |
| **A*_w_*B*_w_*C*_t_*** | (1-s*_p_*)^1/2^ | (1-s*_p_*) | 0 | 0 | 0 | 0 | (1-*s_c_*)^1/2^(1-*s_p_*)^1/2^*H* | (1-*s_c_*)^1/2^(1-*s_p_*) |
| **A*_w_*B*_t_*C*_w_*** | 0 | 0 | 0 | 0 | (1-*s_c_*)^1/2^ | (1-*s_c_*)^1/2^ (1-*s_p_*)^1/2^*H* | (1-*s_c_*) | (1-*s_c_*) (1-*s_p_*)^1/2^*H* |
| **A*_w_*B*_t_*C*_t_*** | 0 | 0 | 0 | 0 | (1-*s_c_*)^1/2^(1-*s_p_*)^1/2^*H* | (1-*s_c_*)^1/2^ (1-*s_p_*) | (1-*s_c_*) (1-*s_p_*)^1/2^*H* | (1-*s_c_*)(1-*s_p_*) |
| **A*_t_*B*_w_*C*_w_*** | 0 | 0 | (1-*s_c_*)^1/2^ | (1-*s_c_*)^1/2^(1-*s_p_*)^1/2^*H* | 0 | 0 | (1-*s_c_*)^1/2^ | (1-*s_c_*)^1/2^(1-*s_p_*)^1/2^*H* |
| **A*_t_*B*_w_*C*_t_*** | 0 | 0 | (1-*s_c_*)^1/2^(1-*s_p_*)^1/2^*H* | (1-*s_c_*)^1/2^(1-*s_p_*) | 0 | 0 | (1-*s_c_*)^1/2^(1-*s_p_*)^1/2^*H* | (1-*s_c_*)^1/2^ (1-*s_p_*) |
| **A*_t_*B*_t_*C*_w_*** | (1-*s_c_*)^1/2^ | (1-*s_c_*)^1/2^(1-*s_p_*)^1/2^*H* | (1-*s_c_*) | (1-*s_c_*) (1-*s_p_*)^1/2^*H* | (1-*s_c_*)^1/2^ | (1-*s_c_*)^1/2^ (1-*s_p_*)^1/2^*H* | (1-*s_c_*) | (1-*s_c_*) (1-*s_p_*)^1/2^*H* |
| **A*_t_*B*_t_*C*_t_*** | (1-*s_c_*)^1/2^(1-*s_p_*)^1/2^*H* | (1-*s_c_*)^1/2^(1-*s_p_*) | (1-*s_c_*)(1-*s_p_*)^1/2^*H* | (1-*s_c_*)(1-*s_p_*) | (1-*s_c_*)^1/2^(1-*s_p_*)^1/2^*H* | (1-*s_c_*)^1/2^ (1-*s_p_*) | (1-*s_c_*)(1-*s_p_*)^1/2^*H* | (1-*s_c_*) (1-*s_p_*) |

We census genotypic and allelic frequencies each generation in the zygotes produced in the target and neighboring populations. The three loci with either a transgenic or a wild-type allele at each can create 27 different diploid genotypes, with their frequencies in the target and neighboring populations given by $x_{i}^{T}$ and $x_{i}^{N}$. These frequencies can be altered by natural selection and homing to give genotypic frequencies in the germlines of adult individuals, $x_{i_{a}}$, which are given by,

$$x_{i_{a}}^{T}=\frac{x_{i}^{T}f\left( i \right)+x_{j}^{T} f\left( j \right)\theta-x_{i}^{T}f\left( i \right) \tau}{\sum_{i} x_{i}^{T}f\left( i \right)}$$

(s1)

$$x_{i_{a}}^{N}=\frac{x_{i}^{N}f\left( i \right)+x_{j}^{N} f\left( j \right)\theta-x_{i}^{N}f\left( i \right) \tau}{\sum_{i} x_{i}^{N}f\left( i \right)}$$

The first term in each numerator of equations (s1) describes the effect of natural selection and of homing efficiency. Here $f\left( x_{i} \right)$ denotes the relative fitness of genotype $i$, as given in Table S1. The remaining two terms in each numerator describe the increase (and decrease) in genotypic frequencies due to homing-based conversion of genotypes in the germline. The second term in each numerator describes the conversion of genotype $j$ into genotype $i$ in the germline due to homing. Here, the modifier *θ* is equal to 1 for genotypes $i$ that are homozygous for the C*_t_* allele and have at least one copy of the B*_t_* allele, and $\theta=0$ otherwise. The label $j$ identifies the genotype that differs from genotype $i$ only in that it is heterozygous at the C locus. The third term in each numerator describes the decrease in the frequency of genotype $i$ due to homing. The modifier $\tau$ is equal to 1 for genotypes $i$ that are heterozygous at the C locus, and carry at least one copy of allele B*_t_*, and $\tau=0$ otherwise. The denominator gives the mean relative fitness for each population.

As described in the main text, we allow the effective immigration rates to become asymmetric if the two populations diverge in size. As adults carry their gametes into the other population through migration, the new genotypic frequencies after migration, $x_{i_{m}}$, are given by

$$x_{i_{m}}^{T}=\frac{x_{i_{a}}^{T}+\mu_{T}x_{i_{a}}^{N}}{\sum_{i} \left( x_{i_{a}}^{T}+\mu_{T}x_{i_{a}}^{N} \right)}$$

(s2)

$$x_{i_{m}}^{N}=\frac{x_{i_{a}}^{N}+\mu_{N}x_{i_{a}}^{T}}{\sum_{i} \left( x_{i_{a}}^{N}+\mu_{N}x_{i_{a}}^{T} \right)}$$

These genotypic frequencies are used to create a random mating pool to give new zygote frequencies after recombination (Mathematica code provided on DRYAD; see References in the main text).

**Section 2: Incorporating a fitness cost of both underdominance constructs**

If both underdominance constructs (alleles A*_t_* and B*_t_*) impose equal fitness cost on transgenic individuals, the total fitness of complete drive homozygotes is reduced by a factor (1-*s_c_*)^2^. This amounts to a considerable reduction in fitness, especially for high values of *s_c_*, resulting in lower drive efficiency.

**Figure S1** **Dynamics of UTH drive with fitness cost for both underdominance constructs:** Colors show mean engineered allele frequency for underdominance (top row) and payload (bottom row) in an isolated target population. The starting frequency of all released individuals (with full drive complement and only underdominance component) is given on the vertical axis in each panel.

**
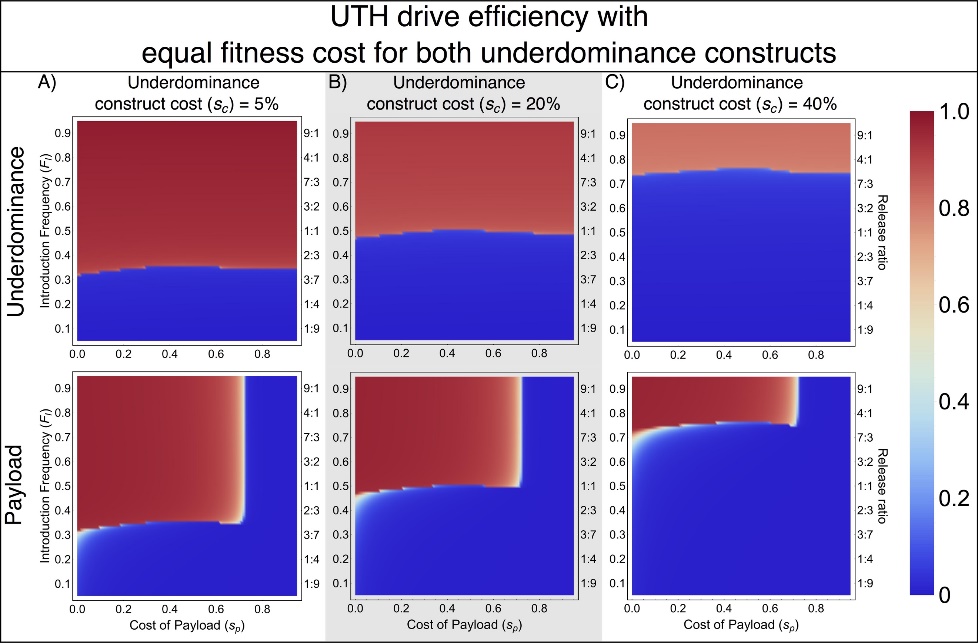
**

**Section 3: Suppression drive with multiplicative payload costs**

A UTH drive intended for population suppression is likely to be more successful when the cost of the payload gene is recessive. A gene drive with a multiplicative cost (Figure S2) result in a lower genetic load than a drive with recessive payload costs (Figure 4).

**Figure S2 Genetic load with multiplicative payload cost** Colors show mean genetic load over twenty generations after drive release (top row) and final genetic load in the twentieth generation (bottom row).


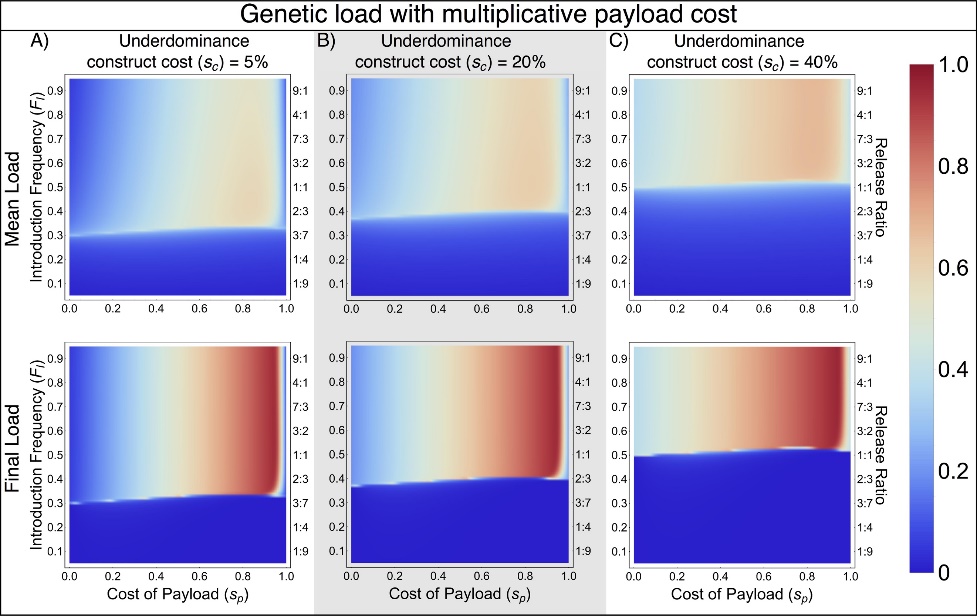


**Section 4: Lower homing efficiency**

Lowering the homing efficiency below 95% (used for all results shown in the main text) reduces the maximum cost of the payload that can still allow successful population alteration. But even with homing efficiency as low as 70%, the UTH drive can be used to establish a payload with almost 50% homozygous fitness cost to both sexes (Figure S3). Payloads with even higher costs can be established if the fitness cost is limited to only one sex.

**Figure S3** **UTH drive dynamics under different homing efficiencies:** Colors show mean engineered allele frequency for underdominance (top row) and payload (bottom row) in an isolated target population. The starting frequency of all released individuals (with full drive complement and only underdominance component) is given on the vertical axis in each panel. *s_c_*=0.05.


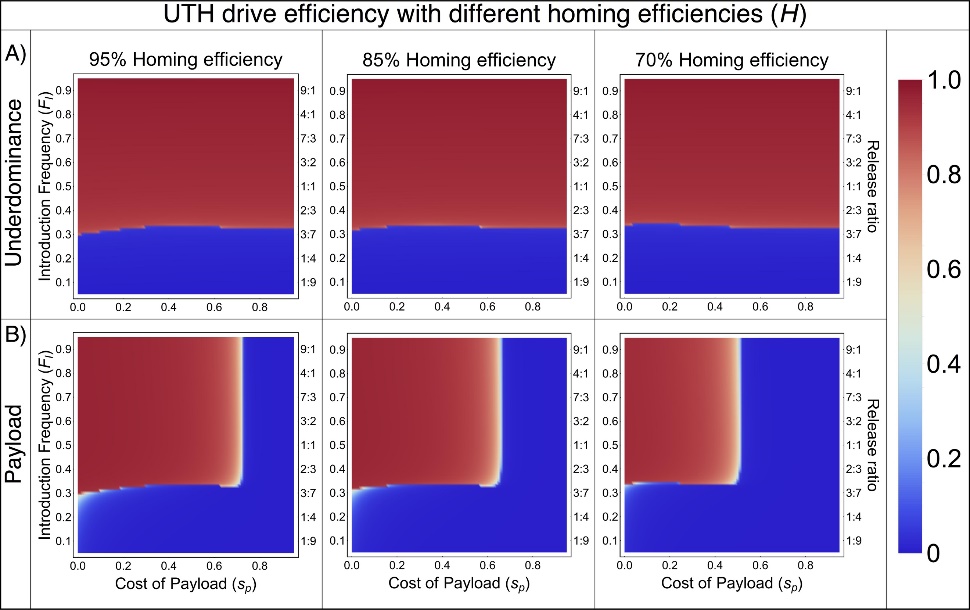


**Section 5: Potential for population suppression with payloads affecting both sexes**

Although a female-limited payload fitness cost can yield a more efficient suppression drive, it may not be easy to limit fitness reduction to only females in some cases. In such cases, sequentially driving multiple payloads with lower costs for both sexes can be used to achieve high genetic loads. For example, in the simulations shown in Figure S4, a UTH drive with a single high cost payload fails to achieve high genetic load, as the payload is lost from the population. However, a similar UTH drive can achieve high genetic load if three payload genes with lower fitness cost are driven sequentially. For comparison, note that a simple two-locus underdominance drive, which can only drive a single payload, is expected to fail completely with the high payload cost and the release sizes used here (Dhole et al. 2018).

**Figure S4: Gradual increase of genetic load with non-sex-specific payload costs:** Time-series plots show frequencies of engineered alleles (top panels) and the gradual buildup of genetic load in the population. The first release has starting frequency of underdominance component at 40% and the first payload at 1%. For the multiple payload scenario, each successive payload is released to achieve 1% starting frequency. Each payload gene in the multiple payload scenario has a 50% homozygous fitness cost affecting both sexes. In the single payload scenario, the payload gene incurs 80% homozygous fitness cost affecting both sexes, and is rapidly eliminated from the population after release. Dashed grey lines show the time of release of successive payloads.


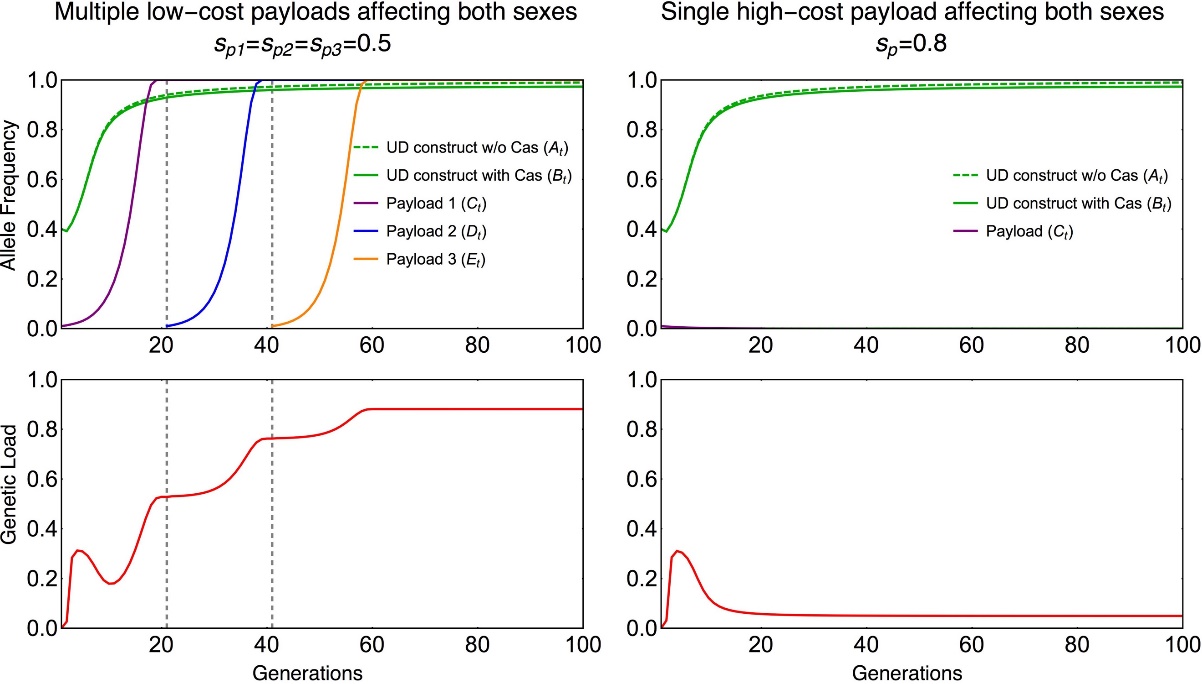


**Section 6: Migration without accounting for changes in adult population size**

The model presented in the main text allows the effective migration rate out of a population to be reduced when the adult population is reduced due to genetic load. This is more realistic than assuming that the same number of individuals migrate out of a population irrespective of changes in the number of individuals in that population. We still show in this section localization results without accounting for such changes (Figure S1).

In a scenario where the effective migration rate is not affected by population size, the wildtype migrants coming into the target population have a smaller impact on the success of a UTH drive. This is because this scenario assumes that the relative contribution of migrants in a population does not change even when the target population has fewer adults compared to the neighboring population. Therefore, the UTH drive is capable of localized alteration over a larger range of migration rates in this scenario compared to the scenario shown in the main text. However, for the same reason, the drive is can spread in both populations with much smaller releases at very high migration rates (Figure S1).

**Figure S5** **UTH localization without correction for population size differences:** Colors show mean frequency of the payload allele over 100 generations in the Target and a Neighbor population. Cost of the underdominance component, *s_c_* = 0.05. Different rows show localization level for different homozygous payload costs. The UTH drive is released to attain starting homing construct frequency of 0.05. The starting frequency of all released individuals (with full drive complement and only underdominance component) is given on the vertical axis in each panel.


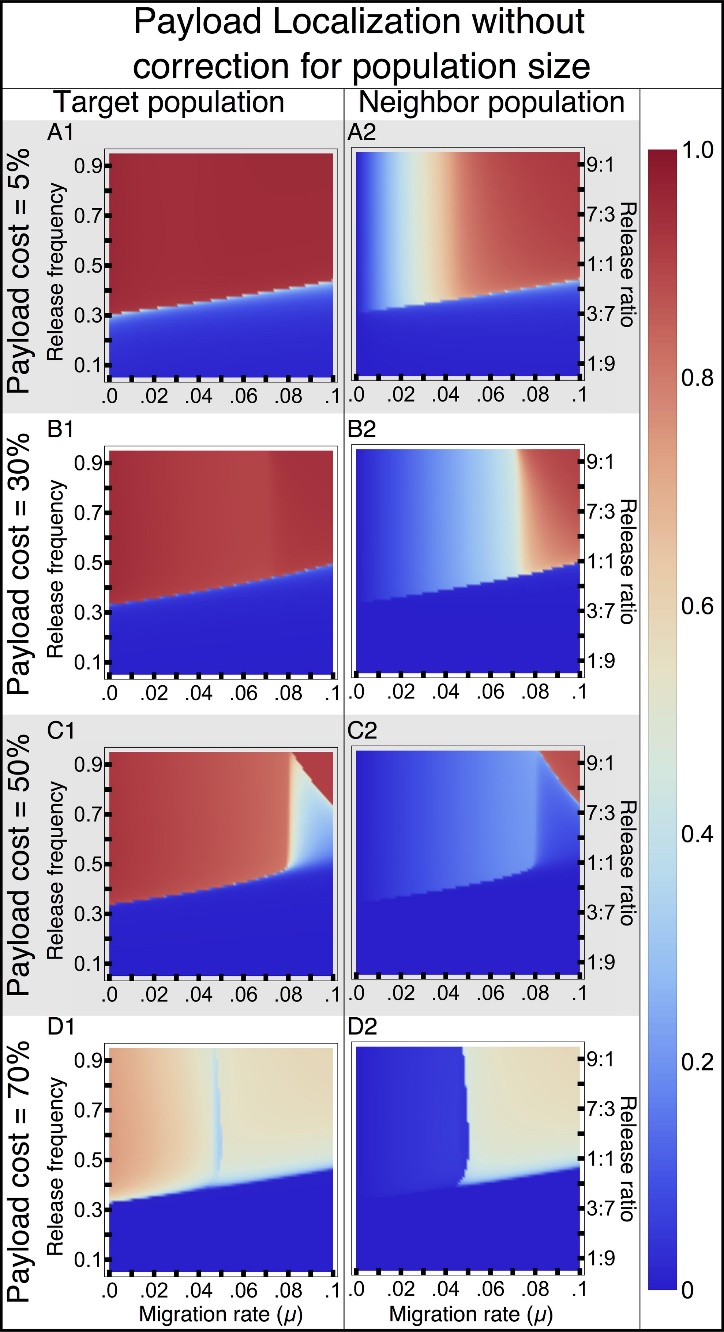
**Section 7: UTH drive localization with higher cost for the underdominance component**

As the cost of the underdominance component increases, the release effort required for successful population alteration also increases. However, higher underdominance costs also result in a more localized gene drive. Even with an underdominance component cost of 20%, the UTH drive has localization level similar to a simple 2-locus engineered underdominance drive.

**Figure S6 UTH localization with costlier underdominance constructs:** Color contours show mean frequency of the payload allele over 100 generations in the two populations for a UTH drive with underdominance component cost (*s_c_*) of 20%. The UTH drive is released to attain starting homing construct frequency of 0.05. The starting frequency of all released individuals (with full drive complement and only underdominance component) is given on the vertical axis in each panel.


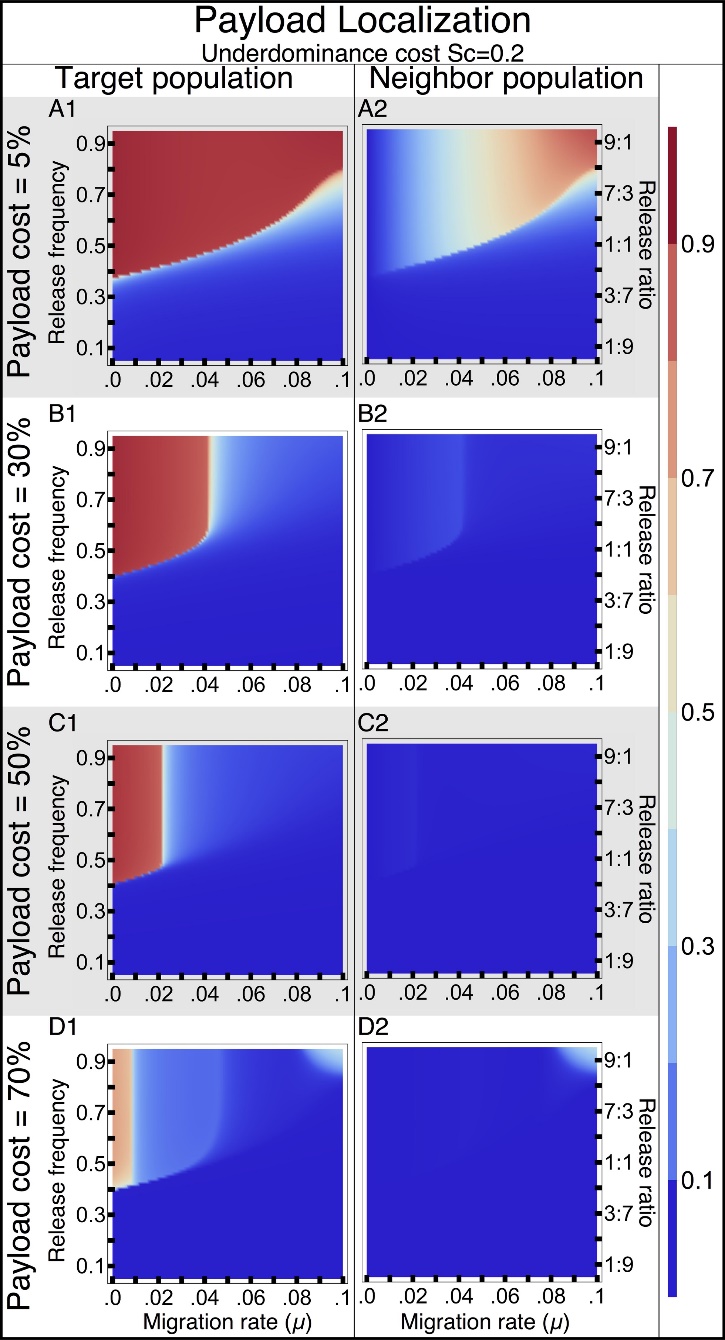


**Section 8: UTH drive with a third toxin gene**

We propose a modified UTH drive with further improved localization. The modification includes the addition of a toxin gene on the homing construct whose transcription is suppressed by a suppressors on the underdominance constructs (Figure S7). This third toxin gene would add a genetic background-specific cost to the homing construct. The addition of the toxin gene improves localization of the payload, especially when the payload gene itself does not impose a high fitness cost (Figure S8A).

**Figure S7** **UTH system with toxin-linked homing construct:** The modified UTH drive includes a toxin gene with a cis-promoter on the homing construct, which is suppressed by a suppressors on the underdominance construct. The toxin is expressed in individuals that possess only the homing construct, rendering them inviable.


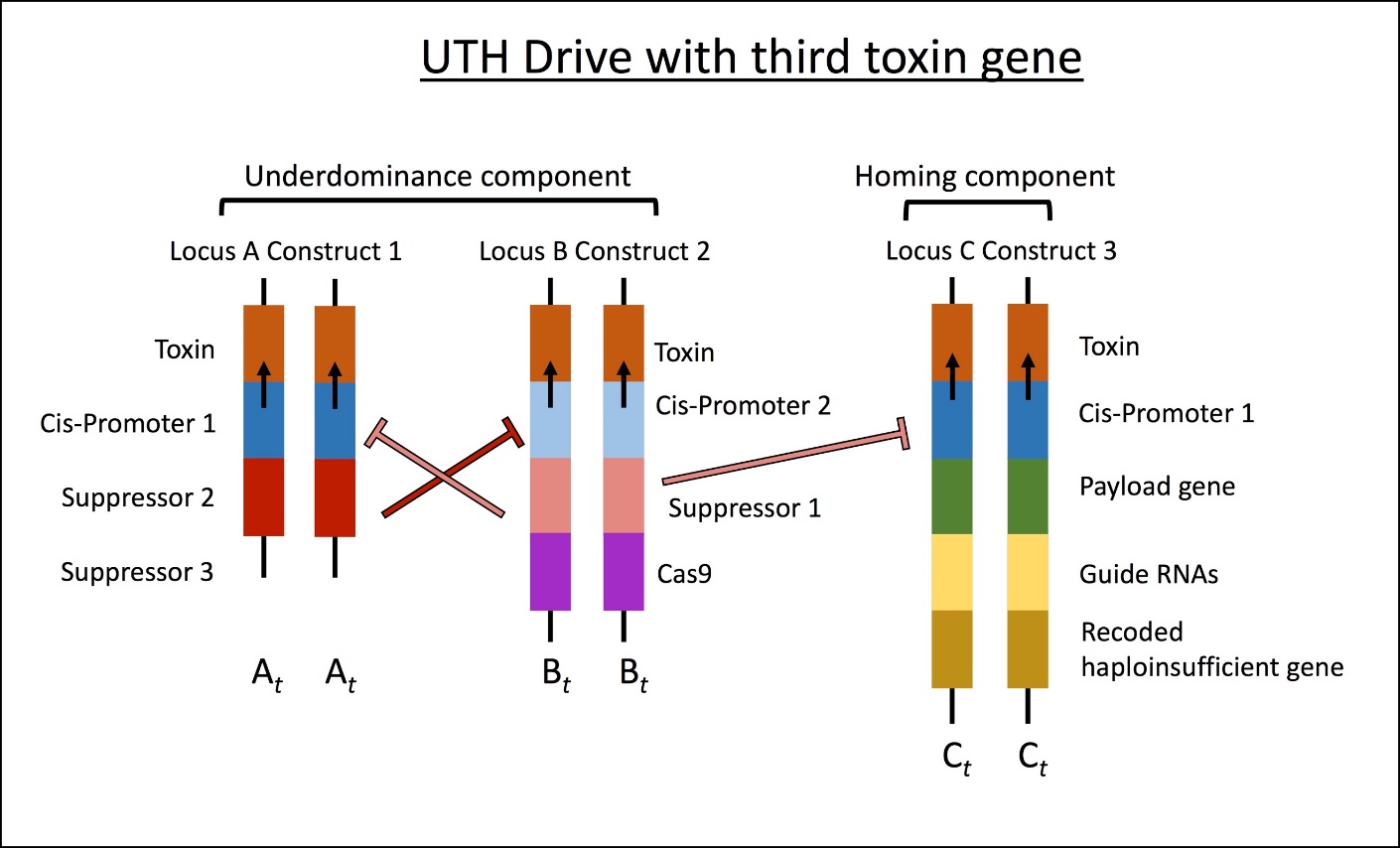


**Figure S8 Payload localization with toxin-linked homing construct:** Color contours show mean frequency of the payload allele over 100 generations in the two populations for a modified UTH drive with an additional toxin gene on the homing construct and underdominance component cost (*s_c_*) of 20%. The drive is released to attain starting homing construct frequency of 0.05. The starting frequency of all released individuals (with full drive complement and only underdominance component) is given on the vertical axis in each panel.


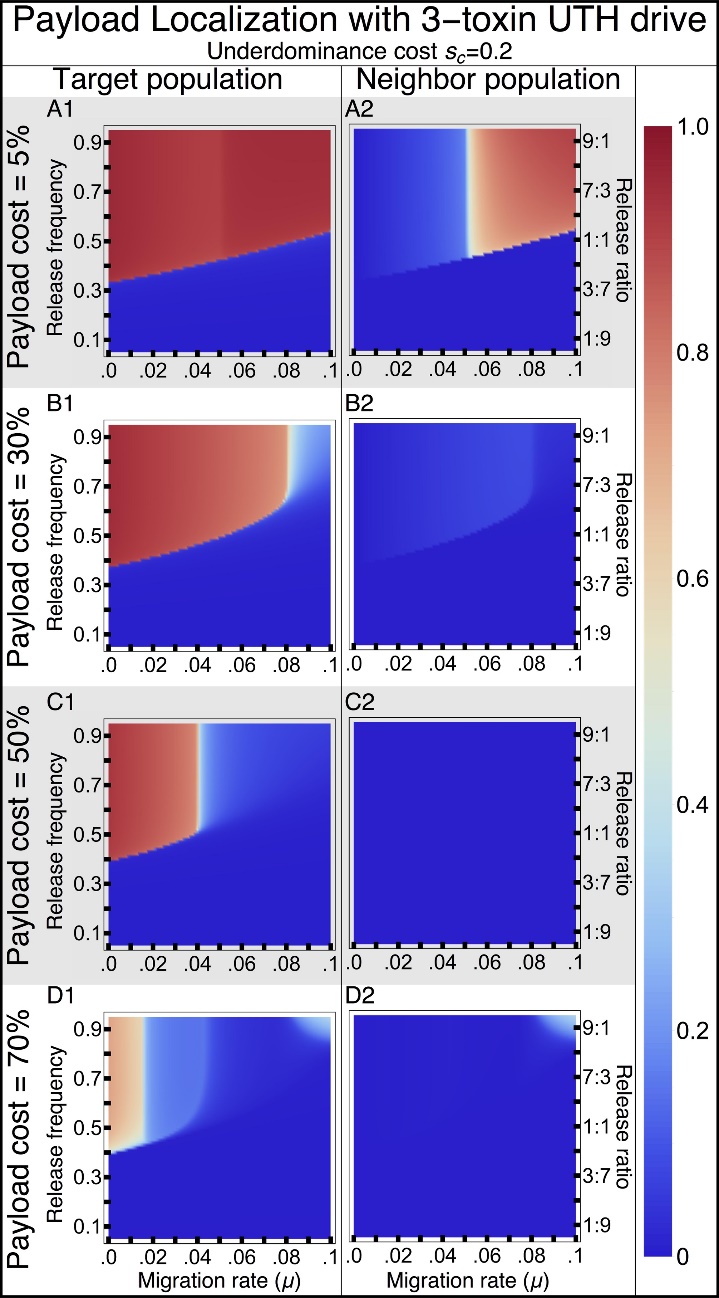


**Section 9: CleaveR-based tethered homing drive**

The recently published Cleave and Rescue gene drive system (Oberhofer et al. 2019) provides a highly feasible method to construct a tethered homing drive (Figure S9). The CleaveR system, which already includes a Cas9 endonuclease, would function as an anchor and would require only the addition of a homing construct. The current construct created by Oberhofer and colleagues is a low threshold system (and thus not highly localized) under laboratory conditions, and may require modifications to function as a strong anchor. As Oberhofer et al. describe, a CleaveR system that targets a haploinsufficient gene would have a higher release threshold and, thus, exhibit a higher localization level. The concept is highly adaptable, and may even be the ideal first choice for creating a tethered homing drive.

**Figure S9 CleaveR Tethered Homing drive:** A modified CleaveR system that targets a haploinsufficient gene can be used as an anchor, where the homing construct would be dependent upon the Cas9 that is located on the CleaveR system.
